# Correction: Modeling of core-shell magneto-electric nanoparticles for biomedical applications: Effect of composition, dimension, and magnetic field features on magnetoelectric response

**DOI:** 10.1371/journal.pone.0314414

**Published:** 2024-11-20

**Authors:** Serena Fiocchi, Emma Chiaramello, Alessandra Marrella, Giulia Suarato, Marta Bonato, Marta Parazzini, Paolo Ravazzani

The images for Figs 1, 2, 4 and 5 are incorrectly switched. The image that appears as Fig 1 should be Fig 4, the image that appears as Fig 2 should be Fig 5, the image that appears as Fig 4 should be Fig 2 and the image that appears as Fig 5 should be Fig 1. The figure captions appear in the correct order.

**Fig 1:**
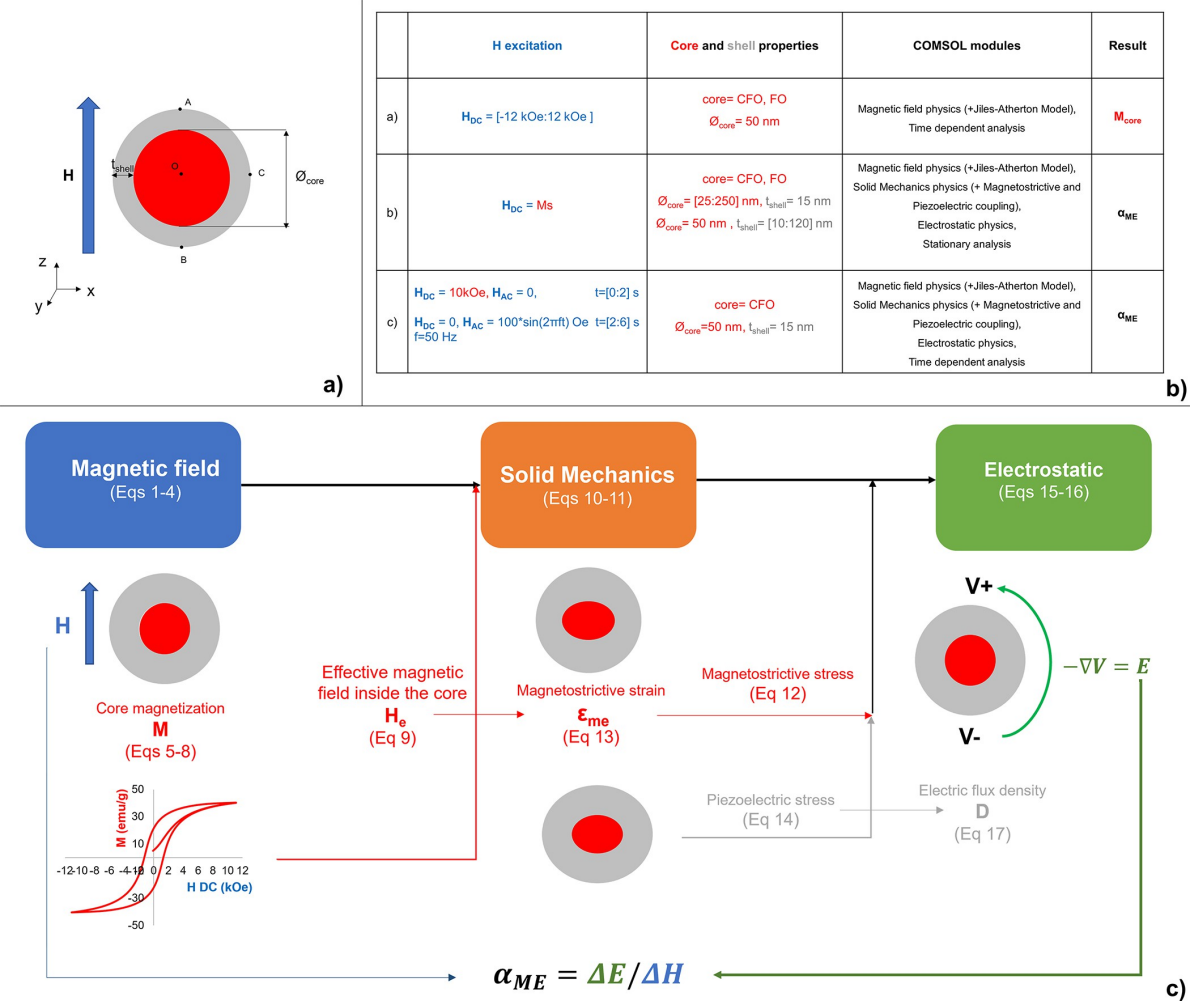
MENP computational modeling. Schematic representation of: a) the geometrical parameters of a generic core-shell MENP; b) the simulation settings in the three different analyses performed; c) the computational study workflow.

**Fig 2:**
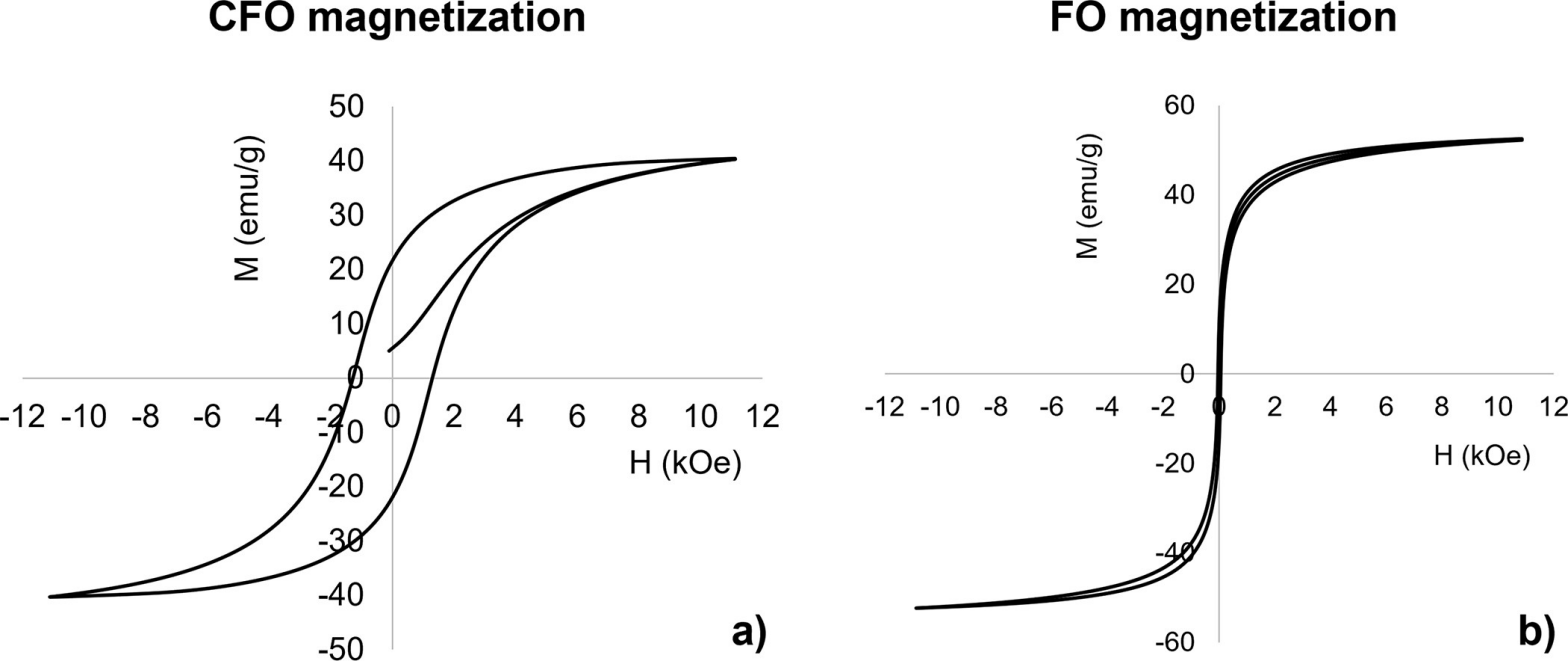
MENPs cores magnetization behavior. DC magnetization loops of a) CFO and b) FO core 50 nm nanoparticles.

**Fig 4:**
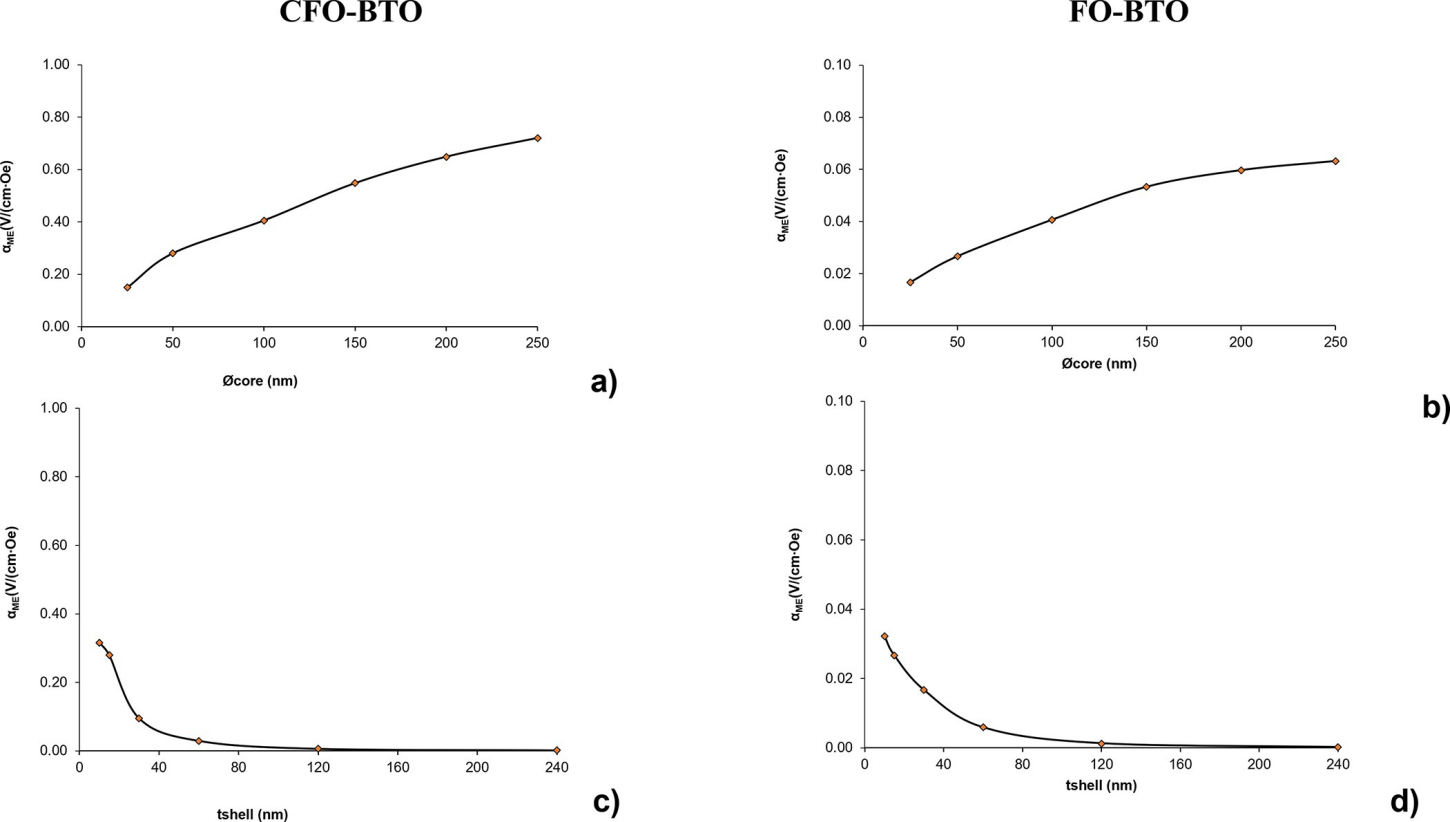
Effect of core and shell size on magnetoelectric coefficient. Trend analysis of variable core size (a and b) and shell thickness (c and d) of CFO-BTO (a and c) and FO-BTO (b and d) MENPs when stimulated with a high strength (> Ms) DC bias magnetic field directed along z on the magnetoelectric coefficient α_ME_ (V/cm∙Oe).

**Fig 5:**
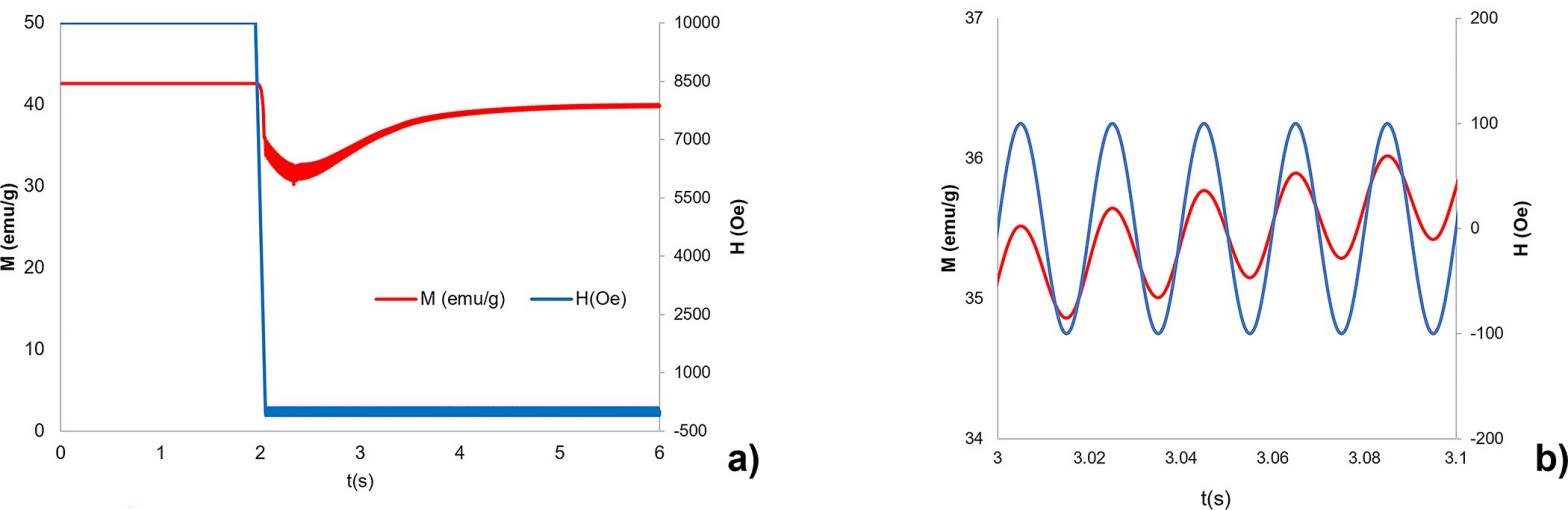
Magnetization of MENP under DC+AC stimulation. Magnetization M(emu/g) (red line) of a CFO core (Ø_core_ = 50 nm)-BTO shell (t_shell_ = 15 nm) nanoparticle under a DC+AC external magnetic field (H (Oe)- blue line) directed along z. a) M(emu/g) as a function of 2 seconds DC high amplitude (H = 10 kOe) magnetic field followed by 4 seconds weak AC (f = 50 Hz, 100 Oe) magnetic field excitation. b) Magnification of Fig 5A in five AC excitation periods.
