# Supplementary material for: Modeling of core-shell magneto-electric nanoparticles for biomedical applications: Effect of composition, dimension, and magnetic field features on magnetoelectric response
Source: PLoS One. 2022 Sep 23;17(9):e0274676. doi: 10.1371/journal.pone.0274676 (PMC9506614; doi:10.1371/journal.pone.0274676)
Supplement: Attachment — Submitted filename: Response to reviewers.docx [file pone.0274676.s002.docx]

|  | Reviewer comment | Response |
| --- | --- | --- |
| 1 | First of all, the Abstract should contain answers to the following question: why is this investigation important? | Thanks for this important comment.  We have now specified in the abstract the reason why this study is important.  “To fully exploit their potentialities and to adapt their use to *in vivo* applications, this study analyzes, through a numerical approach, their magnetoelectric behavior, shortly quantified by the magnetoelectric coupling coefficient (α_ME_), thus providing an important milestone for the characterization of the magnetoelectric effect at the nanoscale.” (page 1, lines 17-20) |
| 2 | The Introduction should make a compelling case for why the magnetoelectric nanoparticles is beneficial with a clear statement of its novelty and originality by providing relevant information and providing answers in connection with nanoelectrodes | In the introductive section, we have added two paragraphs about the advantages of using MENPs in the biomedical field, with particular emphasis on their use as nanoelectrodes in brain stimulation.  See page 2 (lines 26-27) and page 3 (lines 1-12)  “In other words, magneto-electric nanoparticles (MENPs) can wirelessly convert externally applied magnetic field into electric field at the nanoscale and this makes them an extremely innovative and valuable candidate for manipulating electric fields at cellular and subcellular levels. Their use as wireless nanoelectrodes could indeed enable a wide range of applications in which their energy efficiency and the lack of invasiveness represent tremendous advantages over currently used approaches. This is the special case of brain stimulation. It is noteworthy that electric-field triggered stimulation is the basis for many modern stimulation techniques such as invasive direct-contact deep-brain stimulation (DBS) and low-efficacy transcranial magnetic stimulation (TMS). However, these approaches are severely limited in their potentials. The DBS entail direct physical contacts with the brain tissues and is therefore constrained to a finite number of implants. TMS is connected only indirectly with neural networks, and therefore, has very low efficiency and poor spatial resolution. In contrast, wireless ME-based stimulation can be performed locally, and therefore, can be completely non-invasive (or only minimally invasive) while achieving unprecedently high efficacy.  Other possible biomedical applications that can benefit from such innovative and unique nanocomposites include neural recording, nano electroporation, cancer treatment, drug delivery, tissue engineering and imaging (for a complete survey of potential uses in biomedical field see the review studies (11–13)).” |
| 3 | What are the special cases of your study? | As detailed in the previous response, this study aims to model the behavior of MENPs with specific reference to their possible use in biomedical research and then most properties reported in Table 1 and 2 are taken from nanoparticles recently developed for in vitro and in vivo experiments. We have better specified this concept in different parts of the text.  See  Page 3, lines 1-3:  “Their use as wireless nanoelectrodes could indeed enable a wide range of applications in which their energy efficiency and the lack of invasiveness represent tremendous advantages over currently used approaches. This is the special case of brain stimulation.”  Page 5, lines 13-15  “ […] with an eye to their possible application to brain stimulation.  page 6, lines 9-10:  “Most of them are taken from recently synthesized nanoparticles for *in vitro* and *in vivo* experimental studies for brain stimulation and drug delivery (30,36,37)..”  Page 15, lines 3-4  “These theoretical results need however to be framed into the special cases for which specific MENPs are developed.” |
| 4 | Article needs proofreading to eliminate minor typos | Done |
| 5 | Basic equations need to be referenced. | Done |
| 6 | Conclusions should contain an emphasis on the comparison between this work and others for validation. | Thank you for the comment, we have added this concept also in the conclusion:  See page 16, lines 3-6  “The values here found agree with values measured and calculated in experimental studies on MENPs with same properties and stimulated at the same conditions, thus validating our model. Moreover, our results provide additional information that can be strategically used to adapt their use to specific biomedical applications, where safety issues still hamper their performance. “ |
| 7 | Punctuation is missing after some equations. | Done |
| 8 | For enhancing the introduction section with the new publications, old references may be replaced with new ones such as: Dynamism of a hybrid Casson nanofluid with laser radiation and chemical reaction through sinusoidal channels On the entropy optimization of hemodynamic peristaltic pumping of a nanofluid with geometry effects Performance enhancement of a DC-operated micropump with electroosmosis in a hybrid nanofluid: fractional Cattaneo heat flux problem Three-dimensional nanofluid stirring with non-uniform heat source/sink through an elongated sheet Computational Framework of Magnetized MgO–Ni/Water-Based Stagnation Nanoflow Past an Elastic Stretching Surface: Application in Solar Energy Coatings Biomedical simulations of nanoparticles drug delivery to blood hemodynamics in diseased organs: Synovitis problem Leveraging elasticity to uncover the role of rabinowitsch suspension through a wavelike conduit: Consolidated blood suspension application | Thank you for these references.  We have added the suggested and new references in the introduction:  See page 2, lines 13-19  “For example, the investigation and applications of nanoscale liquids, liquid interfaces, and fluids, briefly referred to as nanofluidic (1), has provided revolutionary solutions for handling extremely small volumes of liquids, thus representing a powerful tool for the study of molecular behaviors, intermolecular interactions, and molecule-environment interactions in solution (2–7). Similarly, magnetic nanoparticles have drawn exceptional attention from different technological applications due to their high chemical stability with enhanced surface area, biocompatibility, tunable magnetic moment and functionalization with polymers and other materials (8,9). |
